# Vestibulo-Ocular Responses and Dynamic Visual Acuity During Horizontal Rotation and Translation

**DOI:** 10.3389/fneur.2019.00321

**Published:** 2019-04-09

**Authors:** Cecilia Ramaioli, Luigi F. Cuturi, Stefano Ramat, Nadine Lehnen, Paul R. MacNeilage

**Affiliations:** ^1^German Center for Vertigo and Balance Disorders, University Hospital Munich, Munich, Germany; ^2^Institute of Medical Technology, Brandenburg University of Technology Cottbus-Senftenberg, Senftenberg, Germany; ^3^Department of Psychosomatic Medicine and Psychotherapy, Klinikum Rechts der Isar, Technical University of Munich, Munich, Germany; ^4^Unit for Visually Impaired People, Italian Institute of Technology, Genoa, Italy; ^5^Department of Electrical, Computer and Biomedical Engineering, University of Pavia, Pavia, Italy; ^6^Department of Psychology, Cognitive and Brain Sciences, University of Nevada, Reno, NV, United States

**Keywords:** vestibular system, vestibular ocular reflex, oculomotor, dynamic visual acuity (DVA), otoliths, semicircular canal, retinal slip, eye movements

## Abstract

Dynamic visual acuity (DVA) provides an overall functional measure of visual stabilization performance that depends on the vestibulo-ocular reflex (VOR), but also on other processes, including catch-up saccades and likely visual motion processing. Capturing the efficiency of gaze stabilization against head movement as a whole, it is potentially valuable in the clinical context where assessment of overall patient performance provides an important indication of factors impacting patient participation and quality of life. DVA during head rotation (rDVA) has been assessed previously, but to our knowledge, DVA during horizontal translation (tDVA) has not been measured. tDVA can provide a valuable measure of how otolith, rather than canal, function impacts visual acuity. In addition, comparison of DVA during rotation and translation can shed light on whether common factors are limiting DVA performance in both cases. We therefore measured and compared DVA during both passive head rotations (head impulse test) and translations in the same set of healthy subjects (*n* = 7). In addition to DVA, we computed average VOR gain and retinal slip within and across subjects. We observed that during translation, VOR gain was reduced (VOR during rotation, mean ± SD: position gain = 1.05 ± 0.04, velocity gain = 0.97 ± 0.07; VOR during translation, mean ± SD: position gain = 0.21 ± 0.08, velocity gain = 0.51 ± 0.16), retinal slip was increased, and tDVA was worse than during rotation (average rDVA = 0.32 ± 0.15 logMAR; average tDVA = 0.56 ± 0.09 logMAR, *p* = 0.02). This suggests that reduced VOR gain leads to worse tDVA, as expected. We conclude with speculation about non-oculomotor factors that could vary across individuals and affect performance similarly during both rotation and translation.

## Introduction

During natural movements, head perturbations have both translational and rotational components. In order to compensate for such movements and to maintain a stable image on the retina, the central nervous system (CNS) generates compensatory movements, most notably driven by the vestibulo-ocular reflex (VOR). Depending on the type of head movement, two kinds of VOR are distinguished: the rotational VOR– in response to angular motion sensed by the semicircular canals (SCCs)—and the translational VOR– in response to linear motion sensed by otoliths.

The rotational VOR (rVOR) has been extensively studied. Performance is typically quantified by applying passive rotational head movements [as in the head-impulse test—HIT (1)], measuring eye and head velocity and computing the ratio of the two, which is referred to as the gain. The need for a precise and reliable measure of the oculomotor responses led to the use of video-oculography through head mounted cameras to record eye movements during the HIT (2, 3). Typical movement profiles have a frequency content in the order of 5–7 Hz, characterized by small amplitudes (10°-20°) and peak accelerations of 2,000–7,000°/s^2^ (2, 4). Gain is most often computed during the first 100 ms following movement onset to ensure that responses are driven by vestibular input only. Visually-driven eye movements have a latency of more than ~100 ms, while vestibularly-driven ones have a latency of <10 ms (1, 5). Gains near one are expected in normal subjects; the threshold for clinical diagnosis of pathological VOR response is gain <0.79 (3, 5, 6).

The translational VOR (tVOR) has been much less studied, in part because it can be difficult to administer well-controlled and repeatable passive translation stimuli. Past research has investigated tVOR in response to both horizontal (7–9), and vertical (10–12) translations. Typical movement profiles contain frequencies in the order of 1.5–2 Hz, with peak velocities and accelerations of 25–40 cm/s and 0.7–1 g. As with rVOR, gain is most often computed during the first 100 ms following movement onset to ensure that responses are driven by vestibular input only. Unlike the rVOR, viewing geometry dictates that larger eye movements are needed to stabilize near compared to far images during translational movement, implying that only images lying at the same viewing distance can be stabilized with a single eye movement. Compensation for linear head motion is incomplete, with reported gains between 0.1 and 0.63 with near viewing distances (8, 13). The reason why compensation is incomplete is still a matter of debate. Although linear movements pose less of a threat to stabilization for viewing distances above about 1 m, because of the mentioned inverse relationship with movement amplitude required for stabilization, published results have shown that the gain remains under-compensatory and roughly constant with different viewing distances, hinting that the compensated amount represents a choice of the CNS (7, 8), and not a limitation of the tVOR. In fact, one possible explanation for this finding is that the goal of the tVOR might not be that of stabilizing a single target of interest, but to minimize retinal image motion between objects lying in different depth planes in order to optimize motion parallax information (11, 12).

In addition to quantifying VOR gain, functional visual stabilization performance can be assessed by other techniques, such as measuring dynamic visual acuity (DVA), i.e., the ability to discern fine details of the visual image (14) during both active (15, 16) and passive (17, 18) head motion and different types of visual stimuli (18, 19). Passive head motion is however more informative in the detection of a vestibular dysfunction, as predictive strategies are not available (20, 21). Two further techniques have been proposed for such functional vestibular testing over the last 10 years: the gaze stabilization test (GST) (22, 23) and the functional head impulse test (fHIT) (24–26). All three are based on requiring the patient to identify an optotype displayed on a computer screen during head rotations, yet they differ in terms of visual stimulus triggering criteria and outcome measure: a change in visual acuity measure (logMAR) for the DVA test, the maximum head velocity that does not reduce visual acuity for the GST, the percentage of correctly identified optotypes during head rotations within a range of head angular accelerations for the fHIT. In contrast with VOR gain, functional testing provides a measure of the overall effectiveness of stabilization performance, including not only VOR but also catch-up saccades and other visually-driven responses. Thus, functional testing approaches can provide a clinically valuable measure of overall functional impairment (24, 25, 27–29).

Recent studies have assessed and compared VOR gain and HITD-FT (or functional head impulse test, fHIT) in response to head rotations (30, 31). Administration of opioids led to a decrease in VOR gain and also a decrease in the percentage of correctly identified targets (%CA) during HITD-FT, such that response gain and %CA were significantly correlated (30), yet no correlation was found in a group of patients with vestibular neuritis both on the affected and on the healthy side (29). It was also observed that catch-up saccades performed while the visual target was still present likely led to better reading performance (30, 31) and to better DVA (32).

To our knowledge, no prior study has examined both VOR and DVA in response to pure linear, passive horizontal head movement. The current study therefore aimed to address this gap by measuring both VOR and DVA in response to linear horizontal head movement and comparing these with measures of VOR and DVA during angular head movement in a single group of subjects. We expected to replicate prior findings that VOR gain is reduced during translation compared to rotation, and we expected that reduced gain should lead to DVA that is worse during translation compared to rotation. We also aimed to test the hypothesis that linear and angular measures of VOR and DVA are correlated with one another, which would suggest that performance in response to both linear and angular movements are affected by common factors or mechanisms that are not necessarily vestibular in origin (e.g., visual or perceptual mechanisms).

## Materials and Methods

### Subjects

Seven healthy subjects (4 males), aged 27–41 years (median 33 years) participated in the study. They reported no history of neurological, neuro-otological, or neuro-ophthalmological disorders. Six subjects had normal vision, one subject had vision corrected to normal via glasses. In case subjects normally wore glasses, they performed the task without them because of set-up constraints both for the DVA and the static visual acuity, the latter measured prior to the test (see further in this section). The experimental procedure was approved by the Ethics Committee of the Medical Faculty of the Ludwig-Maximilians-University and in accordance with the Declaration of Helsinki. All gave their informed consent prior to participation and were free to withdraw from the experiment at any time.

### Experimental Procedure: Rotational VOR and DVA

First, the static visual acuity (SVA) was assessed. To this end, subjects were asked to identify 20 fixed sequential visual stimuli displayed on a monitor (size 60 × 53 cm, resolution 1,280 × 800 pixels, refresh rate 75 Hz) connected to the measuring laptop and situated 2 m in front of them, without moving their head. This procedure provided a baseline measure of subjects' visual acuity. The visual stimulus consisted of a Landolt ring with eight possible gap positions at 45° increments. As described in Colagiorgio et al. (33), subjects had to identify the position of the gap and provide answers using an external computer keypad consisting of buttons for each gap position and a special button if they had low confidence in their answer in order to further reduce the possibility of random correct answers. The size of the stimulus was adjusted depending on the subject's error rate, according to the QUEST algorithm (34) performed with the head still, starting from a value of 1 logMAR and estimating subject's acuity threshold in 20 trials, in analogy to the test performed in Colagiorgio et al. (33) to assess SVA.

**Figure 1:**
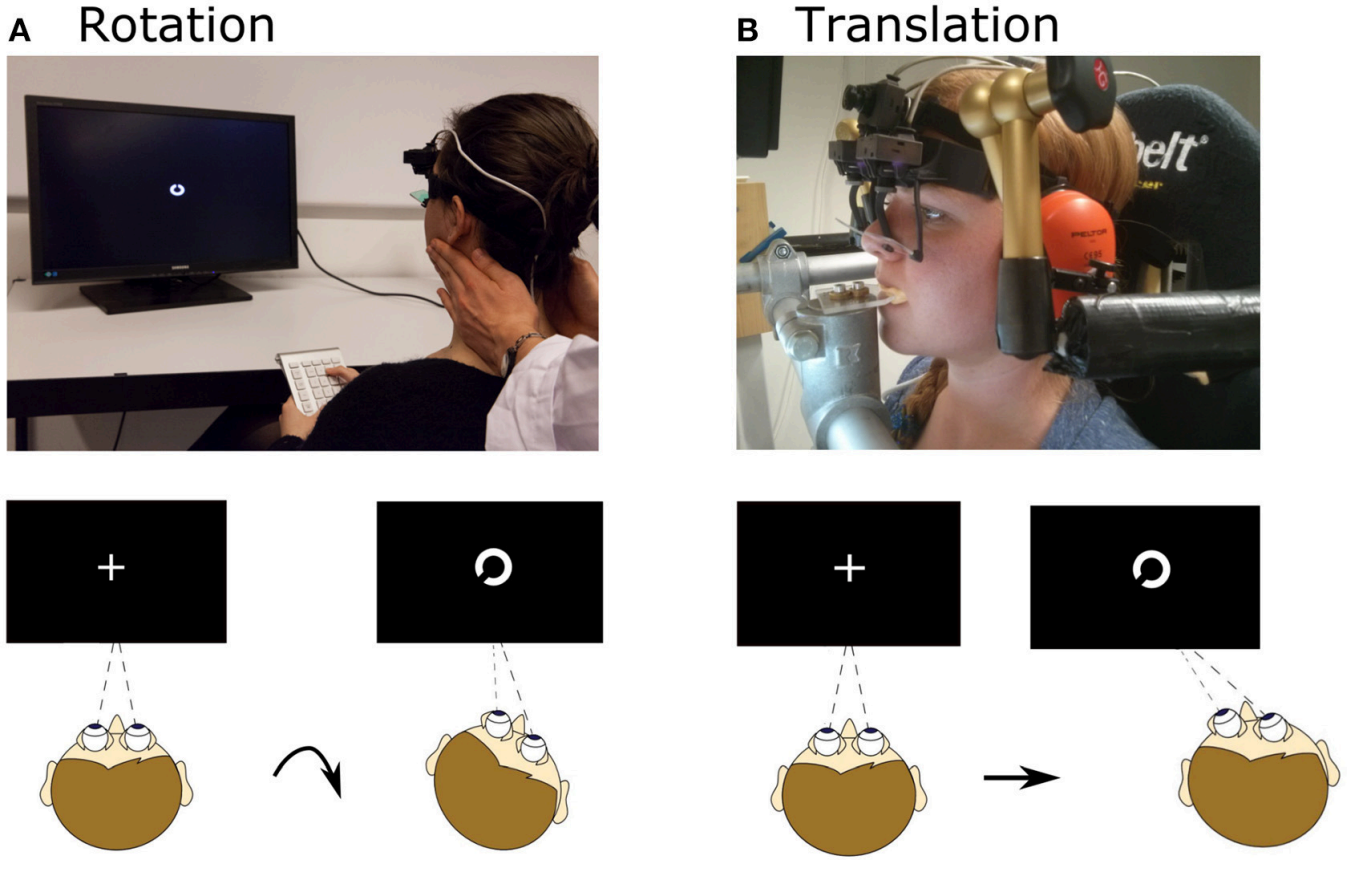
Setup for rotational and translational VOR and DVA assessment. **(A)** Head rotation was induced by a trained experimenter manually rotating the head, as during a clinical head-impulse test. Subjects fixated a fixation point, which changed to a Landolt ring ~72 ms after movement onset and displayed for ~68 ms. After the movement subjects judged the orientation of the ring. **(B)** Translational movements were applied using a six-degree-of-freedom motion platform. The head was fixated with respect to the platform via bite bar and stabilizing braces over the ears. As for rotation, a Landolt ring appeared ~75 ms after movement onset and was displayed for ~49 ms; subjects judged its orientation. Written informed consent was obtained from the individual for the publication of the image represented in the figure.

**Figure 2:**
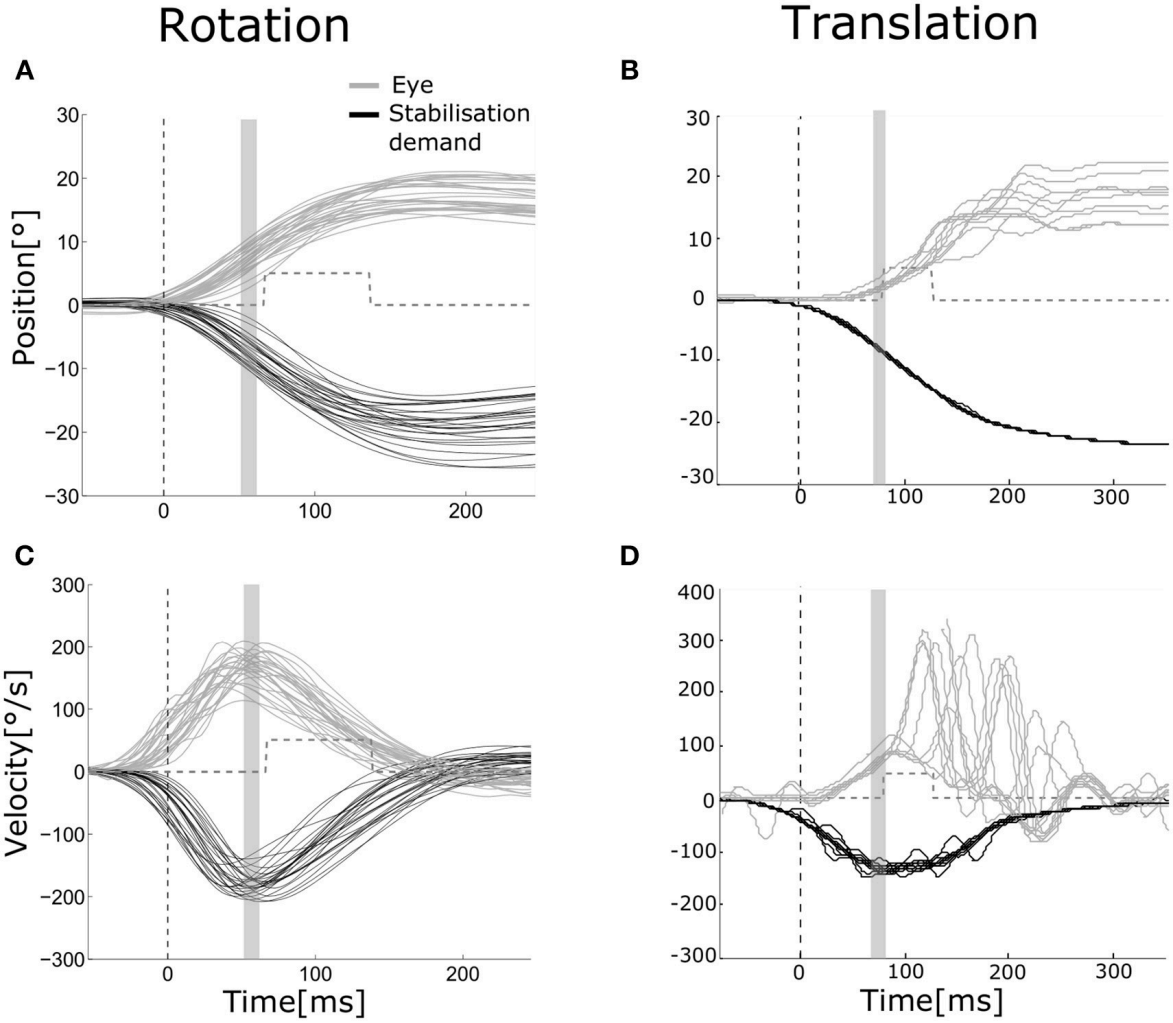
Eye and head movements during rotation and translation Eye movement (gray) plotted vs. stabilization demand (black) for a representative subject during rotation [**(A,C)** head and eye movements to the left and to the right pooled, left eye] and translation [**(B,D)** movements to the right, right eye]. Position **(A,B)** and velocity **(C,D)** traces are aligned to stimulus presentation beginning. The dashed vertical line indicates the mean head movement start. The gray area indicates the time interval over which position and velocity gains of the VOR were computed (55–65 ms after movement onset). The gray dashed line shows the mean time interval when the visual stimulus was turned on to assess dynamic visual acuity.

Rotational VOR (rVOR) and DVA were then assessed (Figure 1A). An experienced examiner standing behind the subject performed at least 40 passive, rapid (6.4 ± 1.5 Hz), high-acceleration (3,300–4,000°/s^2^ peak), and small amplitude (14°-24°) head rotations to the left and to the right (Figure 2, top) in the plane of the horizontal semicircular canals [standard clinical head-impulses (1)]. The impulses, at least 20 to the right, and at least 20 to the left, were delivered with random timing and direction, to prevent anticipatory compensatory movements. Display of the visual stimulus was triggered when head angular acceleration (as measured by the gyroscope integrated in the eye tracker) exceeded 300°/s^2^, otherwise the trial was repeated. The actual timing of the visual stimulus was documented with a photodiode taped to the monitor (33). A fixation cross appeared prior to every rotation in the center of the screen. The visual stimulus was programmed to last 80 ms and it appeared on the screen on average 72 ± 2 ms (mean ± SD) after head movement start (defined as head velocity reaching 20°/s) as recorded with the photodiode. The visual stimulus remained on for 68 ± 3 ms (mean ± SD).

During all testing procedures, eye and head movements were recorded by monocular video-oculography on the left eye and integrated six-degrees-of-freedom inertial sensors [EyeSeeCam system, (2)]. Prior to each testing, calibration of the system was performed following its standard procedure using a laser-projected target grid at 1.5 m viewing distance (6). Sampling rate was 220 Hz.

### Experimental Procedure: Translational VOR and DVA

Translational movements were applied using a six-degree-of-freedom motion platform (Moog^©^ 6DOF2000E). Subjects were seated in a padded racing seat mounted on the platform. In order to guarantee only linear translations in the horizontal plane during the assessment of the DVA, the subject's head was stabilized by means of a bite bar and passive noise canceling headphones connected through mechanical arms to metal poles fixed onto the motion platform (Figure 1B).

Subjects first performed calibration of the system and SVA assessment as previously described. Visual stimuli were projected on a screen (size 45 × 35 cm, resolution 1,400 × 1,500 pixels, refresh rate 75 Hz) located 15 cm in front of subjects' eyes. The screen was mounted to the platform, but the projector (Acer P5403) was mounted to the wall and therefore rendered an earth-fixed visual stimulus. Distance from projector to screen was 73 cm.

After the SVA was completed the translational protocol was performed. Each horizontal linear translation lasted 0.5 s and followed a Gaussian speed profile characterized by a displacement of 8 cm, peak velocity of 0.7 m/s and peak acceleration of 1.3 g (Figure 2, bottom). The delivery of translations, at least 20 toward the left (L) and at least 20 toward the right (R), was computerized with pseudorandom timing and direction, to prevent anticipatory compensatory movements. Onset of the visual stimulus was delayed by a fixed duration relative to the command to move the platform. The actual onset of the visual stimulus was documented with a photodiode taped to the screen as previously described. Onset was 75 ± 5 ms (mean ± SD) after platform movement start (defined as platform displacement of 3 mm from its starting position). A fixation cross appeared prior to every translation, to help subjects maintain the correct vergence angle. In the translation experiment, differently from the rotation experiment, and due to setup constraints, the visual target was projected on a screen (screen size 45 × 35 cm). The projector (Acer P5403, resolution 1,400 × 1,500 pixels, refresh rate 75 Hz) was mounted to the wall and connected to the measuring laptop. As in the rotational experiment, during translations the visual stimulus was programmed to last 80 ms and lasted on average 49 ± 2 ms, likely because of differences in the setup between the two experiments (e.g., the image was presented on a projection screen while in the rotation experiment was presented on a monitor). The difference of stimulus duration between rotations and translations is significantly different (paired *t*-test, *p* < 0.001). Eye and head movements were recorded by binocular video-oculography and integrated six-degrees-of-freedom inertial sensors [EyeSeeCam system, (2)]. Sampling rate was 220 Hz. Platform and head position was also recorded by an optical tracking system at 117 Hz (Optitrack S250e cameras and Motive software).

### Data Analysis

For the rotation experiment, head angular velocity was derived from the sensors mounted on the EyeSeeCam system (2). Eye and head velocity (Figure 2, top) were processed as in Ramaioli et al. (30): eye velocity was filtered with a third order low-pass digital Butterworth filter with a cut-off frequency of 40 Hz, while head velocity was filtered by a second order zero-phase low-pass digital Butterworth filter with 30 Hz cut-off frequency. Eye and head position were computed from velocities. Head impulse start and end were automatically detected when head velocity first reached 20°/s and when it crossed zero again. Impulses with peak velocity slower than 80°/s were discarded. In addition, traces deemed noisy based on visual inspection were also discarded (manual correction). On average, 18 ± 3 head impulses were included in the rVOR analysis for each side. Position gain of the rVOR was computed by taking the median of eye and head positions in a window between 55 and 65 ms after head impulse start, and then taking the ratio of these median values. Velocity gain was computed using the same procedure applied to the velocity traces.

For the translation experiment, trial onset was defined as the moment when the motion platform had moved 3 mm away from its starting position, according to the optical tracking data. Stabilization demand for each trial (Figure 2, bottom) was computed based on viewing distance (v), inter-pupillary distance (ipd), and platform displacement (d) as *atan((–d*± *ipd/2)/v)* and was used to compute the gain of the tVOR as recorded/ideal eye movement (9). This calculation assumes that the platform displacement corresponds to head displacement, i.e., that the head was fixed with respect to the platform. The validity of this assumption was verified by measuring and comparing both head and platform movement. This analysis showed that measures taken to stabilize the head (bite bar and ear cups) were effective, resulting in only small rotations (~2°) of the head relative to the platform. Eye position was filtered with a second order low-pass digital Butterworth filter with a cut-off frequency of 20 Hz. Eye position and stabilization demand were set to zero at the beginning of each trial, with possibility for manually discarding trials showing artifacts or re-fixation saccades in the first 90 ms after movement onset, as they could affect gain calculation. For each eye and direction of movement, 12 ± 3 trials were then considered. Velocities were derived from position traces. Position and velocity gain were computed as described above using data in a time window between 55 and 65 ms after movement onset. Gains were computed separately for the right and the left eye.

During translations, tVOR gain was often low and often catch-up or re-fixation saccades were triggered. Eye movements with an acceleration higher than 2,000°/s^2^ were considered to be re-fixation saccades: saccade onset was defined when acceleration reached 2,000°/s^2^, while saccade offset was defined with acceleration threshold of −2,000°/s^2^. The primary measure of interest was the latency of the first re-fixation saccade after movement onset, because shorter latencies had been suggested to result in better functional performance when VOR gain is reduced (30, 35, 36).

Visual acuity in static (SVA) and dynamic (DVA) condition is tested requiring the subject to identify the orientation of a sequence of 20 Landolt rings. The size of the ring (and its gap) is scaled in accordance to the size and resolution of the screen, and to the subject's viewing distance to correspond to a Sloan eye chart. During testing the optotype size is reduced depending on the subject rate of incorrect answers using the QUEST adaptive algorithm, implemented in the Psychtoolbox. The algorithm starts with a value of 1 logMAR and estimates the subject's visual acuity threshold expressed in units of in logMAR in 20 trials. DVA was calculated as the difference between the threshold value given by the adaptive procedure and the SVA value and was computed separately for rotations to the right and to the left, and for translations to the right and to the left (see Table 1).

**Table 1:** SVA, rDVA, and tDVA data.

**Subject**	**SVA**	**SVA**	**rDVA**	**rDVA**	**tDVA**	**tDVA**
**ID**	**(rotation)**	**(translation)**	**right**	**left**	**right**	**left**
s01	0.00	0.00	0.46	0.49	0.22	0.72
s02	0.00	0.00	0.56	0.46	0.59	0.55
s03	0.06	0.00	0.3	0.36	0.33	0.77
s04	0.00	0.00	0.22	0.36	0.34	0.69
s05	0.00	0.00	0.41	0.29	0.65	0.40
s06	0.63	0.00	0.17	0.09	0.59	0.91
s07	0.03	0.00	0	0.26	0.55	0.59

*Rotation SVA and rDVA were taken with a monitor 2 m away from the subject. Translation SVA and tDVA were taken with a projection screen 15 cm away from the subject. Subject s06 is nearsighted and usually wears glasses as shown by the different SVA values for rotation and translation; however, they did not wear glasses during both SVA and DVA assessment*.

In addition, to examine how VOR gain could impact DVA, we also computed maximum gaze (i.e., head + eye) position and velocity during presentation of the visual stimulus. If this position and velocity are close to zero, the target should be near the fovea and relatively still on the retina, resulting in better acuity. If the target is far from the fovea and moving on the retina, even only a few degrees per seconds, DVA should be impaired and vision deteriorates (37, 38).

All analyses were performed offline using custom MATLAB software (MathWorks, Natick, MA).

### Statistical Analysis

Differences in position and velocity gains during rotations to the right and left were assessed using a *t*-test with significance level of 0.05 (normal distribution verified by Shapiro-Wilk Test). The same procedure was applied to assess differences between gains of the right and left eye during translations to the left and to the right as well as differences in DVA depending on movement direction. As there were no significant differences, data were pooled across eyes and movement directions for all measures and test conditions.

Correlation analysis was performed across both rotational and translational measurements between DVA and all gain and slip measures.

## Results

Examples of eye movements in response to both rotation and translation are shown in Figure 2. The healthy rVOR (Figure 2, left) drives the eye to compensate almost perfectly for the imposed head rotation. Position and velocity gain computed during the time window ~70 ms after movement onset (shown by the gray bar) are very near unity (position gain 1.07 ± 0.07, velocity gain 0.95 ± 0.09). Thanks to this compensation, the subject could correctly identify the visual stimulus when it was present (gray dashed line), achieving an rDVA of 0.29 logMAR (left rotations 0.36, right rotations 0.22), compared to an SVA of 0 logMAR. Data for all subjects regarding rDVA and tDVA as well as SVA are reported in Table 1.

In contrast, eye positions and velocities during translations were insufficient to compensate for head movement. For the example subject shown in Figure 2 (right), position gain pooled across eyes and movement directions was 0.23 ± 0.07 and velocity gain was 0.42 ± 0.12. This decreased gain was associated with reduced tDVA during translation of 0.47 logMAR (0.22 to the right, 0.72 to the left) compared to an SVA of 0 logMAR.

**Figure 3:**
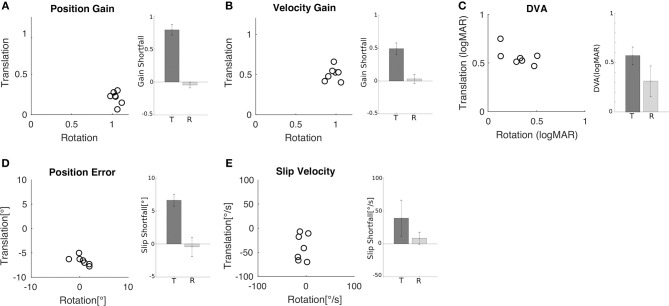
Comparison of rotational and translational VOR gain, slip, and DVA. **(A)** Position error for translation vs. rotation for all subjects. Inset shows the mean (±SD) shortfall in gain relative to gain of one across subjects for translation (0.79 ± 0.08) and rotation (−0.05 ± 0.04). **(B)** Velocity gain for translation vs. rotation for all subjects. Inset shows the mean (±SD) shortfall across subjects for translation (0.49 ± 0.09) and rotation (0.07 ± 0.03). **(C)** DVA for translation vs. rotation for all subjects; larger values indicate worse acuity. Inset shows the mean (±SD) across subjects for translation (0.56 ± 0.09 logMAR) and rotation (0.32 ± 0.15 logMAR). **(D)** Retinal error during translation and rotation for all subjects. Negative values indicate under compensation. Inset shows the mean (±SD) shortfall relative to zero error across subjects for translation (6.61° ± 0.89°) and rotation (−0.44° ± 1.46°). **(E)** Retinal slip velocity during translation and rotation for all subjects. Inset shows the mean (±SD) shortfall across subjects for translation (39 ± 27.2°/s) and rotation (8.57 ± 9.05°/s).

Similar behavior and performance was observed in all seven subjects. Both position gain (Figure 3A) and velocity gain (Figure 3B) were significantly greater during rotation than during translation (paired *t*-test, *p* < 0.001). Mean position gain was 1.05 ± 0.04 during rotation and 0.21 ± 0.08 during translation. Mean velocity gain was 0.97 ± 0.07 during rotation and 0.51 ± 0.16 during translation. The insufficiency of ocular responses during translation is captured by the shortfall relative to a gain of 1 (i.e., 1 minus gain) which is plotted in the inset of Figures 3A,B.

Reduced VOR gains indicate incomplete compensation for head motion and this should be associated with increased retinal slip and worse DVA scores. To examine this relationship more closely, we computed maximum gaze position (i.e., target position relative to the fovea) and velocity (i.e., target velocity on the retina) as the sum of head and eye position and, respectively, head and eye velocity during acuity target presentation. Position error (Figure 3D) and slip velocity (Figure 3E) differed significantly between rotation and translation (position error, *p* = 0.001; slip velocity *p* = 0.03). They were close to zero during rotation but deviated substantially during translation, with negative values indicating slip due to insufficient ocular compensation. To capture this insufficiency, we plot shortfall in slip compensation (slip times minus 1) in the insets of Figures 3D,E. Measures of slip and gain during rotation and translation were not significantly correlated.

These differences in gain and slip were accompanied by differences in DVA (Figure 3C). The minimum angle of resolution was significantly higher during translation (0.56 ± 0.09 logMAR) than during rotation (0.32 ± 0.15 logMAR) (*p* = 0.02), indicating that the ability to recognize the orientation of the optotype was worse during translations in comparison to rotations. Measures of DVA during rotation and translation for an individual subject were not significantly correlated (Figure 3C; *R* = 0.62, *p* = 0.14).

**Figure 4:**
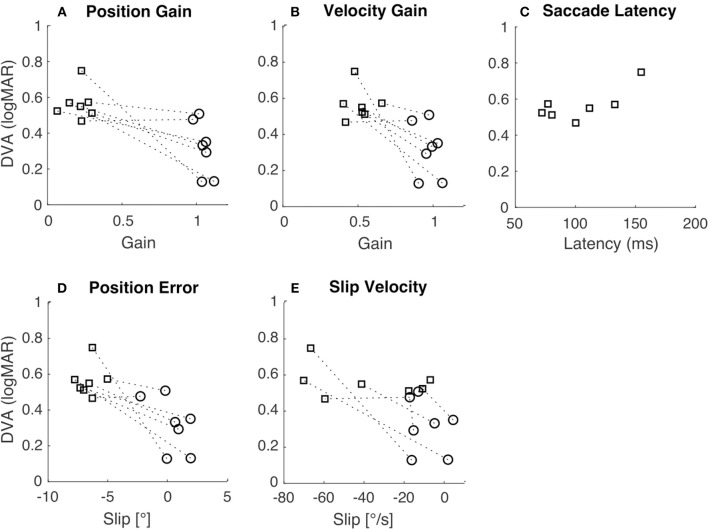
Relation of DVA to gain, slip, and saccade latency. In all panels, squares indicate translation, circles indicate rotation, and the dashed lines connect the two data points from each subject. **(A)** DVA plotted vs. position gain (*R* = −0.75, *p* < 0.01). **(B)** DVA plotted vs. velocity gain (*R* = −0.73, *p* < 0.01). **(C)** DVA plotted vs. saccade latency during translation only (*R* = 0.72, *p* = 0.07). **(D)** DVA plotted vs. position error (*R* = −0.77, *p* < 0.01). **(E)** DVA plotted vs. slip velocity (*R* = −0.59, *p* = 0.03).

To examine how gross differences in gain and slip between rotation and translation contribute to differences in DVA across movement types we tested for correlations between DVA and gain and slip measures. Pooled DVA across movement types was significantly correlated with velocity gain (Figure 4B; *R* = −0.73, *p* < 0.01), position error (Figure 4D; *R* = −0.77, *p* < 0.01), position gain (Figure 4A, *R* = −0.75, *p* < 0.01) and slip velocity (Figure 4E; *R* = −0.59, *p* = 0.03). When correlations were tested using only either rotational or translational data, no significance was observed (Table 2). This suggests that individual differences in either gain or slip do not necessarily allow accurate prediction of DVA performance; other factors are likely to influence DVA performance. We examined one such factor, namely the latency of catch-up or re-fixation saccades. Because gain was low during translation, subjects were often required to make saccades to maintain fixation. Latency of the first such saccade was not correlated with DVA during translation (Figure 4C; *R* = 0.72, *p* = 0.07), despite prior reports that catch-up saccades can play an important role in DVA (30, 31).

**Table 2:** Statistical testing of correlations between DVA and positional gain, velocity gain, position error, and velocity slip.

**Movement type**	**Measure**	***R*-value**	***p*-value**
Rotation	Positional gain	−0.71	0.07
Rotation	Velocity gain	−0.30	0.51
Rotation	Position error	−0.53	0.22
Rotation	Velocity slip	−0.25	0.58
Translation	Positional gain	0.07	0.89
Translation	Velocity gain	0.02	0.97
Translation	Position error	0.18	0.7
Translation	Velocity slip	−0.36	0.43

*DVA during rotational and translational movements was not significantly correlated with gain and slip measures*.

## Discussion

Testing of visual acuity during head movement is important because it provides a functional measure of visual stabilization performance. Historically, the first measures of DVA were performed with a combination of linear and angular vertical head movements [i.e., in the pitch axis (27, 38)]. Several further studies have investigated DVA by focusing on rotational horizontal active movements (15) and also by implementing passive movement techniques to allow unpredictable head rotations (16–18, 28). DVA during translational movements has been most often studied using earth-vertical translations, i.e., movement parallel to gravity, with subjects either upright, such that stimulation was along the vertical axis (10, 39), or on their side, such that stimulation was along the inter-aural axis (40). Here we report, for the first time, a measure of DVA during passive inter-aural translation (tDVA) in the earth horizontal plane. We found that horizontal plane tDVA is worse than rDVA in all subjects, which is in agreement with previous findings of a lower tVOR than rVOR gain (8, 9, 13).

Here, we are particularly interested in measuring and comparing tDVA and rDVA because this comparison allows us to test to what extent acuity is limited by similar factors or mechanisms during translation vs. rotation. Even though ocular responses during rotation and translation are driven by different vestibular organs, the canals and otoliths, respectively, we hypothesized that rDVA and tDVA may be similarly limited by common processes. For example, rVOR and tVOR share the same final common pathway circuitry (i.e., neural integrator, ocular motor plant). In addition, both rDVA and tDVA depend on common visual processes to maintain attention and acuity despite image motion. Several statistical results reported above failed to reach significance, perhaps due to the small number of subjects and lack of statistical power, but we nevertheless offer some speculations below based on these results that may be worthy of further investigation.

### The Impact of VOR Gain and Retinal Slip on DVA

Following conventions in the literature, we computed both positional and velocity gain as the ratio of eye movement to head movement for rotations and the ratio of eye movement to ideal eye movement for translations, in the time window between 55 and 65 ms after movement onset. This ensures that responses are vestibularly-driven because visually-driven responses begin only after 100 ms (1, 5). Observed values for both positional and velocity gain were lower during translation than during rotation (Figures 3A,B), in line with previous reports (3, 7–9, 28). For example, we observed position gains of ~1 for rotational movements whereas translational movements led to positional gains of ~0.20.

We also computed the maximum gaze position error and retinal slip velocity as the sum of the corresponding eye and head quantities. We took the median value during target presentation, which was on average 75 ms after movement onset. Gaze position error during this time interval provides a rough indication of where the target was projected on the retina relative to the fovea. Lower gaze position error values indicate that the target was nearer the fovea, which should result in better acuity. Slip velocity, instead, provides a measure of how the target was moving on the retina during its presentation. Less movement should lead to reduced motion blur, and therefore better visual acuity. We observed greater position error and slip velocity during translation than rotation (Figures 3D,E), as expected based on the reduced gain during translation. These observations are also in line with previous reports (41).

To examine how gain and slip impact DVA, we analyzed the correlation between each of these factors and DVA. We expected that slip, not gain, would be the best predictor of DVA performance because slip provides an absolute measure of position and velocity of the target on the retina, whereas gain is a relative measure. However, we did not observe any significant correlations between DVA and these measures for rotation or translation (Table 2). Nonetheless, a previous study observed a significant correlation between rVOR gain and dynamic reading, but this study considered the functional head impulse testing paradigm instead of the DVA (30).

When similar correlational analyses were performed across pooled rotational and translational measures (Figures 4A,B,D,E) they reached significance. These correlations appear to be driven by gross differences between rotational and translational measures of gain, slip, and DVA. There was considerable variation in DVA measures across individuals, which did not seem to depend on gain or slip. Those subjects with higher rDVA also tended to have higher tDVA, regardless of gain or slip (see dashed lines in Figure 4). The analysis of correlation between rDVA and tDVA (Figure 4C) did not yield a significant result, perhaps due to the limited number of subjects. A significant correlation, which might be observed with greater numbers of subjects in future studies, would indicate that individual differences in factors other than VOR gain and resulting retinal slip contribute to limiting DVA across movement types. Moreover, it should be considered that visual acuity degrades when retinal slip reaches a velocity of ~3°/s (42–44) thus decreasing the potential correlation between retinal slip and DVA.

### The Role of Catch-Up Saccades

When VOR gain is reduced, observers often compensate by making catch-up saccades in order to foveate the target. During our DVA protocol, the target appeared on average after about 72 ms during rotation and after about 78 ms during translation, and then remained on for a period of about 68 ms for rotation and 49 ms for translation. This difference in the stimulus duration can be ascribed to the differences in the set-up used in the two experiments. Clearly, this difference could also have had an influence on our tDVA results both by reducing the time allowed for perception of the optotype and by decreasing the probability that the optotype was on screen at the end of the catch-up saccade. Nonetheless, catch-up saccades executed early enough following movement onset would have resulted in the target landing on or near the fovea before it was extinguished, and this may have allowed for better DVA performance.

We did not typically observe catch-up saccades during rotational movement, probably because VOR gains were close to unity so that these saccades were not necessary. However, during translational movements all subjects exhibited such saccades; examples are shown in Figures 2B,D. To examine the relationship between these saccades and DVA, we computed the average latency of the catch-up saccades for each subject and tested the correlation between this latency and DVA performance (Figure 4C). The correlation was not significant, but we nevertheless observed a trend suggesting that those subjects that executed catch-up saccades with short enough latency were able to partially compensate for the low tVOR gain by moving the target onto the fovea with a saccade before the target was extinguished. This could have allowed these subjects to achieve better DVA despite the low gain and high slip measures. This correlation could also arise (a) because the saccade generally helps with the task, or (b) because those subjects who make early saccades also happen to have better acuity. However, these results are in line with previous studies that observed associations between higher amplitude compensatory saccades with shorter latency and low VOR gain (45, 46) as well as with better HITD-FT performance (31).

### The Possible Role of Non-oculomotor Stabilization Mechanisms

While catch-up saccades can possibly explain improved DVA performance during translation, they do not explain performance during rotation because catch-up saccades were seldom made. Nevertheless, DVA measures during rotation and translation could be related (Figure 3C). In other words, there may be individual differences in DVA performance that persist across movement types. Such an association could arise from non-oculomotor factors limiting DVA performance. For example, one possibility is attention. Subjects who paid greater attention may have performed better in both tDVA and rDVA tasks.

Alternatively, there may be non-oculomotor, visual mechanisms involved in visual stabilization and DVA. Acuity is compromised when (a) the image of the target lands outside the fovea, or (b) the image of the target moves on the retina, resulting in motion blur. However, retinal image motion is not always detrimental to visual acuity. Research on retinal image motion caused by fixational eye movements, including ocular drift and microsaccades, has been extensively studied indicating that visual acuity for high frequency is affected by the absence of fixational eye movements (47). Recent studies show that retinal image motion may actually lead to improved visual acuity compared to the condition in which the retinal image is artificially stabilized using a scanning laser ophthalmoscope (48). This improved acuity is thought to depend on processes that accumulate image information across both space and time to increase the signal-to-noise ratio (49).

If such accumulation processes exist for fixational eye movements, similar processes may operate on a larger spatial scale to augment DVA during the VOR. Indeed, there is evidence of motion deblurring during compensatory eye movements (50). If these mechanisms operate similarly during rotational and translation movement, and there are individual differences in the efficiency of these mechanisms, this could explain an association between tDVA and rDVA that is not accounted for by oculomotor behavior.

To conclude, our study provides a first investigation on how otoliths' function impacts on DVA and found the DVA is consistently lower during translations than during rotations. A more extensive study, involving more subjects and more trials for each subject could clarify the relationship between corrective saccades and functional head stabilization abilities. We suggest that further research on our test accompanied by other specific otolith tests (e.g., oVEMP) and visual acuity assessment procedures might provide a comprehensive picture of the visuo-vestibular interaction underlying translational VOR.

## Author Contributions

CR, LC, PM, and NL conceived the study. CR, LC, PM, and SR designed the experiments and analyzed the data. CR and LC developed the set-up and carried out the experiments. CR, LC, PM, NL and SR wrote and reviewed the manuscript.

### Conflict of Interest Statement

CR was an employee of EyeSeeTec GmbH. NL is a shareholder and paid consultant to EyeSeeTec GmbH. The remaining authors declare that the research was conducted in the absence of any commercial or financial relationships that could be construed as a potential conflict of interest.
